# Association of long-term triglyceride-glucose index patterns with the incidence of chronic kidney disease among non-diabetic population: evidence from a functional community cohort

**DOI:** 10.1186/s12933-023-02098-7

**Published:** 2024-01-03

**Authors:** Ning Chen, Lin-Lin Ma, Yu Zhang, Xi Chu, Jing Dong, Yu-Xiang Yan

**Affiliations:** 1 Department of Epidemiology and Biostatistics, Municipal Key Laboratory of Clinical Epidemiology, School of Public Health, Capital Medical University, Beijing, China; 2 Health Management Center, Xuanwu Hospital, Capital Medical University, Beijing, China; 3 School of Public Health, Capital Medical University, No.10 Xitoutiao, You’anmenWai, Fengtai District, Beijing, 100069 China

**Keywords:** Triglyceride-glucose index, Chronic kidney disease, Variability, Cumulative exposure, Cohort study

## Abstract

**Background:**

The triglyceride-glucose (TyG) index is a reliable surrogate marker of insulin resistance and previous studies have confirmed the association of TyG index with incident chronic kidney disease (CKD). However, the impact of longitudinal patterns of TyG index on CKD risk among non-diabetic population is still unknown. Therefore, this study aimed to investigate the association of longitudinal patterns of TyG index with incident CKD among non-diabetic population.

**Methods:**

A total of 5484 non-diabetic participants who underwent one health examination per year from 2015 to 2017 were included in this prospective study. TyG index variability and cumulative TyG index were calculated to assess the longitudinal patterns of TyG index. Cox proportional hazard models were performed to estimate the association of TyG index variability or cumulative TyG index with incident CKD.

**Results:**

During a median of 3.82 years follow-up, 879 participants developed CKD. Compared with participants in the lowest quartile, the hazard ratio (HR) and 95% confidence interval (CI) of incident CKD were 1.772 (95% CI: 1.453, 2.162) for the highest TyG index variability quartile and 2.091 (95% CI: 1.646, 2.655) for the highest cumulative TyG index quartile in the fully adjusted models. The best discrimination and reclassification improvement were observed after adding baseline TyG, TyG index variability and cumulative TyG index to the clinical risk model for CKD.

**Conclusions:**

Both TyG index variability and cumulative TyG index can independently predict incident CKD among non-diabetic population. Monitoring longitudinal patterns of TyG index may assist with prediction and prevention of incident CKD.

**Supplementary Information:**

The online version contains supplementary material available at 10.1186/s12933-023-02098-7.

## Background

Chronic kidney disease (CKD) has received increased attention as a leading public health problem in the past decades [1]. According to Global Burden of Disease (GBD), CKD was ranked as the 12th leading cause of death among 133 diseases [2]. Besides, renal failure, mortality and cardiovascular events, are all strongly affected by kidney involvement [3, 4]. Thus, it is essential to reduce the incidence of CKD and the burden caused by poor prognosis through early diagnosis and intervention.

Insulin resistance (IR) is a state in which physiologic concentrations of insulin produce a subnormal biologic response [5]. Many studies showed that IR played a critical role in the occurrence and development of CKD [6, 7]. The hyperinsulinemic-euglycemic clamp test is the golden-standard way of assessing IR but as this method is time-consuming and laborious, it is not suitable for routine large-scale clinical investigations [8]. Currently, triglyceride glucose (TyG) index, which was calculated using fasting triglyceride (TG) and fasting blood glucose (FBG), was regarded to be a simple and reliable surrogate index for IR [9, 10]. Recent studies have confirmed the positive association between TyG index and the incidence of CKD [7, 11, 12]. However, TG and FBG levels can be affected by a variety of factors such as air pollution, diet and anxiety [13–15], which in turn leads to fluctuations and changes in the TyG index over time. Therefore, compared to a single measurement, multiple repeated measurements of TyG index could provide a more accurate representation of long-term TyG index patterns over time and may be more significantly associated with adverse outcomes. Some studies have examined repeated measurements of TyG and found that longitudinal TyG index, both in terms of visit-to-visit variability and cumulative exposure, could significantly increase the risk of adverse outcomes [16, 17]. However, as for CKD, most studies focused on a single measurement and few studies have evaluated the impact of longitudinal TyG index on the risk of CKD. Besides, few studies focused on general non-diabetic population.

Considering the different effects of metabolic risk factors in different population and adverse lifestyle, environmental and psychological factors in the diabetic population could contribute to the increased vulnerability to incident CKD and thus have an impact on our results, this study assessed the association of TyG index variability or cumulative TyG index with the incidence of CKD among non-diabetic population and further determined whether these two indices have synergy on incident CKD.

## Methods

### Study design and participants

The present study was performed based on a prospective cohort design and the aim was to evaluate the association of TyG index variability or cumulative TyG index with the incidence of CKD among non-diabetic population. Participants who underwent annual health examination from 2015 to 2017 (baseline and index year) were included and long-term TyG index patterns from three health examination were calculated as predictors of future CKD. All the participants were annually followed under same identical conditions for the development of CKD until December 31, 2021.

The population data used in this study were obtained from the Beijing Functional Community Cohort, which was initiated in 2010 and 8671 individuals with annual examination were recruited at the Health Management Center of Xuanwu Hospital, Capital Medical University [18]. These participants were from various occupations in Xicheng District, Beijing, including medical workers, teaching staff, government workers, workers, and service industries, representing most of the occupational population in Beijing. This cohort excluded participants with a history of medication for elevated blood glucose and dyslipidaemia. Those with acute inflammatory disease, hepatic or renal failure, heart failure, autoimmune disease or cancer were also excluded. The study protocol was approved by the ethics committee of Capital Medical University and Xuanwu Hospital, and it was conducted according to the principles of the Declaration of Helsinki. All study participants were informed at enrollment.

In this analysis, as the collection of information on the cohort population was more limited prior to 2015, we included participants who underwent one health examination per year between January 2015 and December 2017. Of 6759 participants, we excluded those with with missing data on FBG or lipid in 2015, 2016 or 2017 wave (n = 286) and those who were treated with antidiabetic or lipid-lowering agents (n = 371). We further excluded those with a history of type 2 diabetes (T2D) or CKD in 2017. Finally, 5484 participants were included in our analysis and were annually followed under same identical conditions for the development of CKD until December 31, 2021. Fig. S1 in Additional file 1 showed the flow chart of this prospective cohort study and Table S1 in Additional file 1 presented the baseline characteristics of participants included and excluded from this study.

### Clinical measurements

Venous blood samples were collected between 7:30 and 8:30 am after an overnight fast. In a calm state, all samples were immediately centrifuged for laboratory measurements. Low-density lipoprotein cholesterol (LDL-C), high-density lipoprotein cholesterol (HDL-C), TG, alanine aminotransferase (ALT), aspartate aminotransferase (AST), Urea, creatinine (Cr) and uric acid (UA) were measured using standard laboratory methods (Hitachi Autoanalyzer 7060; Hitachi, Tokyo, Japan). FBG was determined by the glucose oxidase method. Hemoglobin A1c (HbA1c) levels were measured by Ion-exchange chromatography. T2D was defined as FBG ≥ 126 mg/dL or HbA1c ≥ 6.5% or self-reported history of T2D.

### Calculation of TyG index, TyG index variability and cumulative TyG index

The TyG index was measured ln (fasting TG [mg/dL]×FPG [mg/dL]/2). TyG index variability was defined as intraindividual variability recorded in the health examinations from 2015 to 2017 and coefficient of variation (CV) was first used to determine the variability of TyG [17, 19]. Standard deviation (SD), average real variability (ARV) and variability independent of the mean (VIM) were also used to determine the variability of TyG in sensitivity analysis and detailed information was presented in Supplementary Materials.

As previous studies described [20, 21], cumulative TyG index was defined as the summation of the average TyG index for each pair of consecutive examinations multiplied by the time between these two consecutive visits, in years: ([TyG index_visit 1_+TyG index_visit 2_]/2×time_1 − 2_) + ([TyG index_visit 2_+TyG index_visit 3_]/2× time_2 − 3_).

TyG index_visit 1_, TyG index_visit 2_, TyG index_visit 3_ indicate the TyG index at the first, second and third examinations, respectively, and time_1–2_, time_2–3_ indicate the participant-specific time intervals between consecutive visits in years.

### Definition of outcome

In this study, CKD was defined as estimate glomerular fltration rate (eGFR) < 60 ml/min/1.73 m^2^ or onset of macroalbuminuria (UACR > 300 mg/g) according to Kidney Disease: Improving Global Outcomes (KDIGO) guidelines [22]. Besides, self-reported diagnosis of CKD was also included in the diagnostic criteria for CKD in this study.

eGFR was calculated based on Chronic Kidney Disease Epidemiology Collaboration (CKD-EPI) [23]. The sex specific formula is as follows:

Women: if Cr ≤ 0.7 mg/dL, eGFR = 144*(Cr/0.7)^−0.329^*(0.993)^Age^.

if Cr > 0.7 mg/dL, eGFR = 144*(Cr/0.7)^−1.209^*(0.993)^Age^.

Men: if Cr ≤ 0.9 mg/dL, eGFR = 141*(Cr/0.9)^−0.411^*(0.993)^Age^.

if Cr > 0.9 mg/dL, eGFR = 141*(Cr/0.9)^−1.209^*(0.993)^Age^.

### Covariates definition

Factors associated with outcomes were considered as covariates. Based on previous studies, covariates in this study included age, sex, current smoking, current drinking, educational level, physical activity, hypertension, BMI, LDL-C, HDL-C, ALT, AST, Cr, UA, Urea, eGFR and baseline TyG index.

Structured standard questionnaires were used to collect basic information about the study participants, including sex, age, current smoking, current drinking, education level, and physical activity. Current smoking was defined as those who smoked ≥ 1 cigarette per day. Current drinking was defined as the intake of wine/beer/cider/spirits ≥ 1 time per week. Physical activity was defined as walking or cycling ≥ 15 min per day, exercising or physical activity > 2 h per week or lifting or carrying heavy loads at work per day.

Height and weight were measured while the participants wearing light clothing without shoes, and body mass index (BMI) was calculated as weight in kilograms divided by the square of height in meters (kg/m^2^). Blood pressure (BP) was averaged after three consecutive measurements using a standard mercury sphygmomanometer on the right arm of the study subject after 5 min of sitting still. Hypertension was defined as systolic blood pressure (SBP) ≥ 140 mmHg or diastolic blood pressure (DBP) ≥ 90 mmHg or self-reported history of hypertension.

### Statistical analysis

Categorical variables were presented as number (proportions), continuous variables were presented as mean ± SD or median (quartiles). Difference between groups were compared using Chi-square test for categorical variables and t test, One-way ANOVA or Kruskal-Wallis rank sum test for continuous variables, as appropriate.

Intraclass correlation coefficient (ICC) was first calculated to assess the stability and consistency of repeated TyG index measurements and if the ICC is greater than 0.75, it indicates a high stability of the repeated measurements. Multivariable-adjusted Cox proportional hazards regression models were used to estimate the association of quartiles of baseline TyG index, TyG index variability or cumulative TyG index with incident CKD and hazard ratios (HR) and 95% confidence intervals (CI) were listed. Model 1 was adjusted for age and sex. Model 2 was further adjusted for current smoking, current drinking, educational level, physical activity, hypertension, BMI, LDL-C, HDL-C, ALT, AST, Cr, UA, Urea and eGFR based on Model (1) Model 3 was further adjusted for baseline TyG index based on Model (2) Time to first CKD event was examined using Kaplan-Meier survival curves and compared using Log-rank test. Restricted cubic spline (RCS) was conducted to exploit the dose-response relationship between TyG index variability or cumulative TyG index and the incidence of CKD. We further divided participants into four groups according to median of TyG index variability and median of cumulative TyG index and compared the risks of CKD among different groups. The relative excess risk due to interaction (RERI) and the attributable proportion due to interaction (AP) were also calculated to assess the synergy. If 0 is outside the 95% CIs of RERI and AP, there are additive interactions of TyG index variability and cumulative TyG index on the incidence of CKD. C statistics, net reclassification improvement (NRI) and integrated discrimination improvement (IDI) were used to estimate the improvement in discrimination and reclassification after adding baseline TyG index, TyG index variability and cumulative TyG index to the clinical risk model for CKD prediction [24].

Multiple sensitivity analyses were performed to validate the robustness and reliability of our results. First, participants experiencing CKD within one year were excluded to minimize potential reverse causality. Second, to account for the possible changes in TyG index levels before index year, the mean values of TyG from 2015 to 2017 instead of baseline values was adjusted in Model 3 of the Cox proportional-hazards model. Third, considering the possible influence of abnormal TG concentrations on TyG index levels or outcomes, restricted analysis was performed by excluding participants with baseline TG concentrations ≥ 1.7 mmol/L. Besides, SD, VIM and ARV were further used to determine the variability of TyG index and the primary analysis was repeated. The potential effect modification by age, sex, BMI and hypertension categories was also evaluated using stratified analysis and interaction testing using a likelihood ratio test.

Statistical analyses were performed using R software (version: 4.2.1; R Foundation for Statistical Computing) and MedCalc® Statistical Software (version 20.100; MedCalc Software Ltd, Ostend, Belgium; https://www.medcalc.org; 2022). The difference was considered statistically significant at two-side significance level of *P* < 0.05.

## Results

Finally, 5484 participants were included in this study and Table 1 showed the baseline characteristics of these participants. Among them, the mean age at the baseline was 52.49 ± 14.67 years and 2486 (45.33%) were men. The median TyG index variability was 0.026 (interquartile range: 0.016–0.037), the median cumulative TyG index was 17.05 (interquartile range: 16.21–17.88). The ICC value was 0.768 (95% CI: 0.759, 0.777) (Table S2 in Additional file 1), which showed the stability of repeated TyG measurements in this study and the importance of utilizing repeated measurements instead of relying solely on single measurement in our study. Table S3 and S4 in Additional file 1 presented baseline characteristics of these participants according to quartiles of TyG index variability and cumulative TyG index. During a median of 3.82 years follow-up, 879 participants developed CKD. Participants who developed CKD were slightly older, current smokers and alcohol drinkers. They also had higher prevalence of hypertension and had higher median BMI, SBP, UA, Glu, TG, baseline TyG index, TyG index variability and cumulative TyG index. Fig. S2 in Additional file 1 showed the incidence of CKD according to quartiles of TyG index variability or cumulative TyG index and participants in higher quartiles of TyG index variability or cumulative TyG index showed significantly higher incidence of CKD (*P* < 0.001).

**Table 1:** Baseline characteristics of study participants

	Total N = 5484	CKD N = 879	Non-CKD N = 4605	*P*
Age, y	52.49 ± 14.67	55.51 ± 14.75	51.91 ± 14.59	< 0.001
Men, n (%)	2486 (45.33)	434 (49.37)	2052 (44.56)	0.009
Smoking, n (%)	343 (6.25)	57 (6.48)	286 (6.21)	0.759
Drinking, n (%)	673 (12.27)	109 (12.40)	564 (12.25)	0.899
Physical activity, n (%)	3982 (72.61)	642 (73.04)	3340 (72.53)	0.757
Education, n (%)				0.206
Primary school and below	155 (2.83)	17 (1.93)	138 (3.00)	
Junior high school	1442 (26.29)	229 (26.05)	1213 (26.34)	
High school and above	3887 (70.88)	633 (72.01)	3254 (70.66)	
Hypertension, n (%)	1306 (23.81)	266 (30.26)	1040 (22.58)	< 0.001
SBP, mmHg	123 [112, 136]	127 [116, 139]	122 [111, 135]	< 0.001
DBP, mmHg	75 [68, 82]	77 [70, 84]	75 [68, 82]	< 0.001
BMI, kg/m^2^	24.22 [22.03, 26.56]	24.83 [22.63, 27.08]	24.11 [21.90, 26.42]	< 0.001
AST, U/L	19 [14, 26]	19 [15, 27]	19 [14, 26]	0.002
ALT, U/L	21 [18, 25]	22 [19, 25]	21 [18, 25]	0.002
TG, mg/dL	114.29 [80.63, 163.25]	128.47 [91.26, 182.07]	111.64 [78.85, 159.48]	< 0.001
LDL-C, mg/dL	112.89 [93.17, 134.25]	115.98 [95.88, 139.18]	112.11 [92.78, 132.99]	0.002
HDL-C, mg/dL	54.90 [46.01, 64.95]	53.35 [44.07, 63.02]	54.90 [46.39, 64.95]	< 0.001
Glu, mg/dL	93.78 [88.38, 100.08]	95.58 [90.00, 102.87]	93.42 [88.02, 99.72]	< 0.001
HbA1c, %	5.3 [5.0, 5.6]	5.4 [5.1, 5.7]	5.3 [5.0, 5.6]	< 0.001
Urea, mmol/L	4.91 [4.15, 5.85]	5.20 [4.31, 6.09]	4.87 [4.13, 5.79]	< 0.001
UA, µmol/L	314 [263, 374]	324 [274, 380]	312 [262, 372]	0.001
Cr, mg/dL	0.72 [0.63, 0.85]	0.75 [0.64, 0.87]	0.72 [0.62, 0.85]	< 0.001
eGFR, ml/min/1.73 m^2^	102.23 [93.16, 111.15]	99.22 [90.88, 107.95]	102.88 [93.80, 111.58]	< 0.001
Baseline TyG	8.60 [8.23, 8.97]	8.72 [8.37, 9.11]	8.57 [8.20, 8.93]	< 0.001
Cumulative TyG index	17.05 [16.21, 17.88]	17.41 [16.68, 18.20]	16.97 [16.13, 17.82]	< 0.001
TyG index variability	0.026 [0.016, 0.037]	0.029 [0.019, 0.040]	0.025 [0.016, 0.037]	< 0.001

CKD: chronic kidney disease, SBP: systolic blood pressure, DBP: diastolic blood pressure, BMI: body mass index, TG: triglyceride; AST: aspartate aminotransferase; ALT: alanine aminotransferase; Cr: creatinine; UA: uric acid; Glu: plasma glucose, LDL-C: low-density lipoprotein cholesterol, HDL-C: high-density lipoprotein cholesterol, TyG index: triglyceride glucose index

**Fig. 1:**
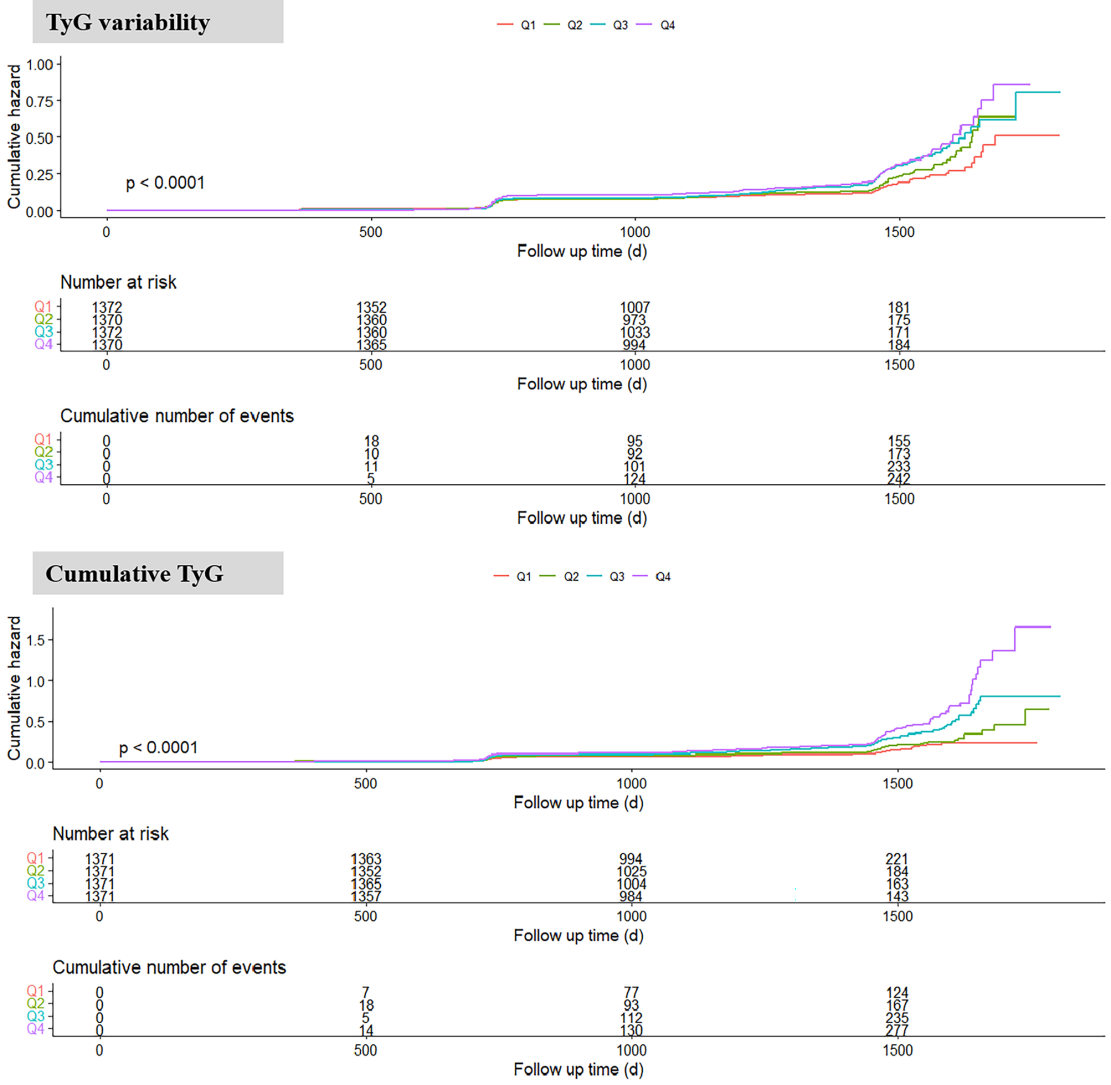
Kaplan-Meier curves of incidence of chronic kidney disease according to different quartiles of TyG index variability or cumulative TyG index

Table 2 presented the association of baseline TyG index, TyG index variability and cumulative TyG index with incident CKD. In Model 2, the baseline TyG index, TyG index variability and cumulative TyG index were all significantly associated with the incidence of CKD. Compared with participants in the lowest quartile (Q1), the HRs of incident CKD in the highest baseline TyG quartile, TyG index variability quartile and cumulative TyG index quartile were 1.572 (95% CI: 1.227, 2.015), 1.772 (95% CI: 1.453, 2.162) and 2.091 (95% CI: 1.646, 2.655), respectively. After further adjusted for baseline TyG index, risks of incident CKD of participants in higher quartiles of TyG index variability and cumulative TyG index also significantly increased compared to those in the lowest quartile (*P* for trend < 0.001). Kaplan-Meier curve showed similar results and participants in higher quartiles of TyG index variability and cumulative TyG index had higher risks of developing CKD (*P* < 0.001) (Fig. 1).

**Table 2:** Association of baseline, variability, cumulative of TyG index with the incidence of CKD

	Model 1		Model 2		Model 3		
	HR (95% CI)	*P*	HR (95% CI)	*P*	HR (95% CI)	*P*	
**Baseline TyG index**							
Q1	-		-		-		
Q2	1.246 (1.009, 1.538)	0.041	1.172 (0.937, 1.465)	0.164	-		
Q3	1.384 (1.125, 1.702)	0.002	1.303 (1.034, 1.642)	0.025	-		
Q4	1.811 (1.486, 2.206)	< 0.001	1.572 (1.227, 2.015)	< 0.001	-		
*P* for trend		< 0.001		< 0.001			
**TyG index variability**							
Q1	-				-		
Q2	1.199 (0.976, 1.474)	0.084	1.177 (0.951, 1.456)	0.134	1.179 (0.953, 1.459)	0.129	
Q3	1.537 (1.264, 1.869)	< 0.001	1.491 (1.219, 1.822)	< 0.001	1.488 (1.217, 1.819)	< 0.001	
Q4	1.757 (1.447, 2.135)	< 0.001	1.772 (1.453, 2.162)	< 0.001	1.714 (1.403, 2.094)	< 0.001	
*P* for trend		< 0.001		< 0.001		< 0.001	
**Cumulative TyG index**							
Q1	-				-		
Q2	1.195 (0.953, 1.498)	0.122	1.144 (0.906, 1.445)	0.258	1.138 (0.899, 1.442)	0.282	
Q3	1.732 (1.400, 2.142)	< 0.001	1.634 (1.299, 2.056)	< 0.001	1.617 (1.268, 2.061)	< 0.001	
Q4	2.206 (1.795, 2.711)	< 0.001	2.091 (1.646, 2.655)	< 0.001	2.049 (1.549, 2.711)	< 0.001	
*P* for trend		< 0.001		< 0.001		< 0.001	

CKD: chronic kidney disease, TyG index: triglyceride glucose index

Model 1 was adjusted for age and sex

Model 2 was additionally adjusted for current smoking, current drinking, educational level, physical activity, hypertension, BMI, LDL-C, HDL-C, ALT, AST, Cr, UA, Urea and baseline eGFR

Model 3 was additionally adjusted for baseline TyG index

The dose-response impacts on CKD of TyG index variability and cumulative TyG index were showed in Fig. S3 in Additional file 1. Both TyG index variability and cumulative TyG index reported significant dose-response relationships on incident CKD (*P* < 0.001) and nonlinear test were significant (TyG index variability: *P* = 0.003, cumulative TyG index: *P* = 0.048). The estimated HRs of incident CKD increased with higher TyG index variability and cumulative TyG index from the linear spline model (*P* < 0.001).

As Table 3 presented, we further divided participants into four groups according to median of TyG index variability and cumulative TyG index. We found compared to the group with both low TyG index variability and cumulative TyG index, those with both high TyG index variability and cumulative TyG index showed highest risk of incident CKD, with HR of 2.298 (95% CI: 1.820, 2.903). Besides, significant additive interactions of TyG index variability and cumulative TyG index on the incidence of CKD were found (RERI: 0.42, 95% CI: 0.23, 0.60; AP: 0.20, 95% CI: 0.11, 0.29).

**Table 3:** Combined association of TyG index variability and cumulative TyG index with the incidence of CKD

	Low cumulative TyG index HR (95% CI)	High cumulative TyG index HR (95% CI)	RERI (95% CI)	AP (95% CI)
Low TyG index variability	-	1.574 (1.233, 2.010)	0.42 (0.23, 0.60)	0.20 (0.11, 0.29)
High TyG index variability	1.463 (1.162, 1.843)	2.298 (1.820, 2.903)		

CKD: chronic kidney disease, TyG index: triglyceride glucose index, RERI: relative excess risk due to interaction; AP: the attributable proportion due to interaction

As was shown in Table 4, the addition of baseline TyG index to clinical models could increase the C-statistic for CKD prediction although it was not statistical significant. Further addition of TyG index variability or cumulative TyG index to the clinical model to which baseline TyG index has been added can significantly improve the C-statistics. Significant reclassification improvement were also observed. After adding baseline TyG, TyG index variability and cumulative TyG index to the clinical models for CKD prediction, the best discrimination and reclassification improvement were observed.

**Table 4:** Reclassification and discrimination statistics for predicting CKD by adding baseline TyG index, TyG index variability and cumulative TyG index

	C-statistic	*P*	NRI	*P*	IDI	*P*
**Men**						
Clinical risk model ^a^	0.610 (0.590, 0.629)					
+baseline TyG index	0.610 (0.590, 0.630)	0.690 ^b^	-0.130 (-0.236, -0.025)	0.015 ^b^	-0.17% (-0.36%, 0.01%)	0.060 ^b^
+baseline TyG index and TyG index variability	0.633 (0.614, 0.653)	0.008 ^c^	0.240 (0.135, 0.345)	< 0.001 ^c^	0.85% (0.36%, 1.35%)	< 0.001 ^c^
+baseline TyG index and cumulative TyG index	0.629 (0.609, 0.648)	0.006 ^c^	0.097 (-0.009, 0.202)	0.072 ^c^	0.42% (0.02%, 0.81%)	0.039 ^c^
+baseline TyG index, TyG index variability and cumulative TyG index	0.649 (0.629, 0.668)	< 0.001 ^c^	0.309 (0.204, 0.414)	< 0.001 ^c^	1.50% (0.88%, 2.12%)	< 0.001 ^c^
		0.007 ^d^	0.088 (-0.017, 0.194)	0.101 ^d^	0.42% (0.00%, 0.83%)	0.048 ^d^
		0.010 ^e^	0.243 (0.138, 0.348)	< 0.001 ^e^	0.95% (0.45%, 1.45%)	< 0.001 ^e^
**Women**						
Clinical risk model ^a^	0.612 (0.593, 0.630)					
+baseline TyG index	0.615 (0.597, 0.633)	0.318 ^b^	0.143 (0.039, 0.246)	0.007 ^b^	0.17% (0.02%, 0.32%)	0.027 ^b^
+baseline TyG index and TyG index variability	0.629 (0.611, 0.647)	0.019 ^c^	0.189 (0.084, 0.294)	< 0.001 ^c^	0.38% (0.12%, 0.64%)	0.004 ^c^
+baseline TyG index and cumulative TyG index	0.623 (0.605, 0.641)	0.107 ^c^	0.156 (0.051, 0.261)	0.004 ^c^	0.22% (0.02%, 0.42%)	0.035 ^c^
+baseline TyG index, TyG index variability and cumulative TyG index	0.636 (0.617, 0.653)	0.004 ^c^	0.233 (0.129, 0.338)	< 0.001 ^c^	0.64% (0.29%, 0.99%)	< 0.001 ^c^
		0.152 ^d^	0.178 (0.073, 0.283)	< 0.001 ^d^	0.26% (0.04%, 0.48%)	0.023 ^d^
		0.026 ^e^	0.192 (0.087, 0.297)	< 0.001 ^e^	0.42% (0.14%, 0.70%)	0.003 ^e^

CKD: chronic kidney disease, TyG: triglyceride-glucose, NRI: net reclassification improvement, IDI: integrated discrimination improvement

^a^ The clinical risk model for incident CKD include age, SBP, DBP, WC, FBG, serum gamma-glutamyl transferase, ALT, AST, TC, HDL-C, LDL-C (only for men), TG, HbA1c, eGFR, BMI, alcohol intake, smoking status, physical activity, past medical history (heart disease, stroke, hypertension, diabetes mellitus (only for women), and hyperlipidemia), and family history (heart disease, stroke, and hypertension). When no information of predictors was available in this cohort, a fixed value for the “missing variables” (i.e., “0” for categorial variables and a fixed number for continuous variables) was used for analysis

^b^ Compared to clinical risk model

^c^ Compared to clinical model with baseline TyG index added

^d^ Compared to clinical model with baseline TyG index and TyG index variability added

^e^ Compared to clinical model with baseline TyG index and cumulative TyG index added

**Fig. 2:**
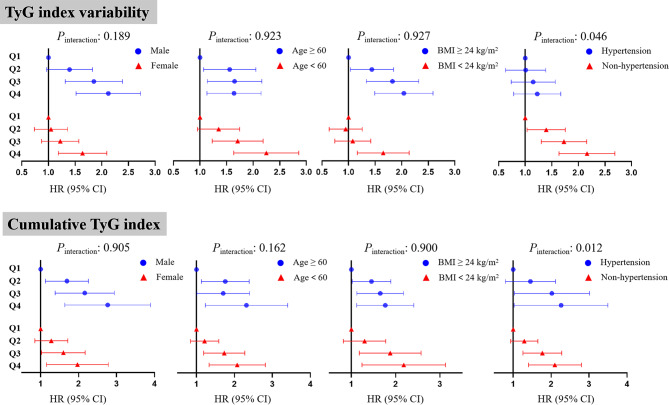
Subgroup analyses: association of TyG index variability or cumulative TyG index with incident chronic kidney disease

In sensitivity analyses (Table S5 and S6 in Additional file 1), our results all remained consist and participants in higher quartiles of TyG index variability or cumulative TyG index had a higher risk of developing CKD (*P* for trend < 0.001). As Fig. 2 showed, subgroup analyses stratified by sex, age, BMI and hypertension were performed and we did not find significant interaction between quartiles of TyG index variability or cumulative TyG index and age (< 60, ≥ 60), sex (men, women) or BMI (< 24, ≥ 24 kg/m^2^). Significant interaction was observed in hypertension and non-hypertension participants (TyG index variability: *P*_interaction_=0.046, cumulative TyG index: *P*_interaction_=0.012). The significant association of TyG index variability or cumulative TyG index with the incidence of CKD were mainly found in non-hypertension participants.

## Discussion

In this study, we selected non-diabetic participants who had one health examination per year from 2015 to 2017 and during a median of 3.82 years follow-up, 16.03% participants developed CKD. We found longitudinal TyG index, both in terms of visit-to-visit variability and cumulative exposure, could significantly increase the risk of incident CKD. Risks of incident CKD of participants in higher quartiles of TyG index variability or cumulative TyG index significantly increased compared to those in the lowest quartile (*P* for trend < 0.001) and sensitivity analyses validated the robustness of our results. We further divided participants into four groups according to median of TyG index variability and median of cumulative TyG index and found the group with both high TyG index variability and cumulative TyG index had the highest risk of developing CKD. After adding baseline TyG, TyG index variability and cumulative TyG index to the clinical models for incident CKD prediction, the best discrimination and reclassification improvement were observed.

Several studies have confirmed the association of TyG index with the incidence of CKD, but subgroup analysis according to the status of diabetes showed different results. Among diabetic population, such association remained significant in most relevant studies. However, among non-diabetic population, Li et al. and Shi et al. still found significant association of TyG index and incident CKD [11, 25], while Gao et al., Liu et al. and Zhu et al. did not find that TyG index can increase the risk of CKD among non-diabetic population [12, 26, 27]. The use of hypoglycaemic drugs in diabetic populations can have an effect on FBG levels and further affect TyG index [28]. Besides, adverse lifestyle, environmental and psychological factors in the diabetic population could contribute to the increased vulnerability to incident CKD. Considering the above factors and the inconsistency of the association of TyG index and incident CKD in non-diabetic populations, this study aimed to assess whether TyG index was the risk factor of incident CKD among non-diabetic population and significant association were found between baseline TyG index and incident CKD.

As researches processed, growing evidence have showed the importance of long-term monitoring of TyG index and the association of long-term TyG index with adverse outcomes has been generally confirmed. A study conducted in China found that high change of TyG index change could significantly increase SBP and DBP (*P* < 0.05) [29]. Another Chinese study showed that the HR for the highest quartile cumulative TyG index group versus the lowest quartile group were 1.59 (95% CI, 1.17–2.16), 1.97 (95% CI, 1.19–3.26), and 1.66 (95% CI, 1.02–2.70) for overall major adverse cardiovascular events, CVD death, and non-fatal myocardial infarctions, respectively [16]. As for incident CKD, no evidence existed. To our knowledge, this is the first study to assess the association of long-term TyG index with incidence of CKD and our study confirmed that both TyG index variability and cumulative TyG index could significantly increase the risk of incident CKD among non-diabetic population and independently of baseline TyG index.

CKD progresses very quickly and can lead to fatal diseases such as kidney failure [30]. However, as the progression process of CKD is not easily detectable, early prediction of this disease is particularly important [31]. Currently, studies have developed different risk predictive models for incident CKD and all showed good performance on CKD prediction [32, 33]. A latest sex-specific predictive model for the development of CKD showed good performance and Harrell’s C of the developed prediction models were 0.82 for men and 0.79 for women [24]. Our study extended TyG index variability or cumulative TyG index to this sex-specific model and the highest incremental effect was observed after extending both TyG index variability and cumulative TyG index to the prediction model, which highlighted the importance of long-term monitoring of TyG index. Today, handheld and mobile technologies essentially put continuous monitoring devices in people’s pockets, and many health systems record all information electronically [34]. Participants’ electronic medical records provide great convenience and feasibility for rapidly integrating data across multiple time points and calculating their long-term variability and cumulative exposure, so as to provide a compelling rationale for using routinely collected electronic health information to assess the long-term impact of risk factors [16].

The mechanisms underlying the relationship between cumulative TyG index and incident CKD remained unclear, but some explanations could be suggested. First, IR was positively associated with elevated levels of inflammatory markers [35] and many studies have confirmed inflammation as an independent risk factor of incident CKD [36]. Besides, releases of proinflammatory cytokines such as interleukin (IL)-6 and tumor necrosis factor (TNF)-α could induce endothelial dysfunction, which was also associated with incident CKD [37, 38]. Second is oxidative stress. IR can activate the mitochondrial electron transport chain, induce excessive production of reactive oxidative stress (ROS), and further lead to kidney tissue fibrosis [7]. Third, hyperinsulinemia has been reported to affect renal function by inducing glomerular hyperfiltration, endothelial dysfunction and increased vascular permeability [39]. Thus, a higher cumulative TyG index means a longer period of exposing to high TyG and further more severe damage to kidney function. As for TyG index variability, a study found that changes in IR may have effects on inflammation and endothelial function, which might be risk factors of incident CKD [40]. Changes in TyG index have also been found to be associated with increased BP, which might play an important role in this association [29]. Besides, we hypothesized that the underlying metabolic dysfunction leads to changes in biomarker levels, altered lipid exchange and catabolism may all have impacts on the development of CKD [41]. It is noted that further studies are still needed to elucidate the mechanisms underlying the association of TyG index variability with increased risk of CKD.

The strength of this study included that it was the first prospective study to investigate the association of longitudinal pattern of TyG index and incident CKD among non-diabetic population. Using longitudinally and repeatedly collected TyG data at the individual level before the incidence of CKD, visit-to-visit variability and cumulative exposure were both assessed. Besides, all available confounders were adjusted and different sensitivity analyses were performed. However, our study also has some limitations. First, as this was an observational study, the causal relationship of TyG index variability or cumulative TyG index with incident CKD could not be fully demonstrated. Second, selection of study population based on the number of health examinations could be subject to selection bias. Finally, participants in this study were limited to adults in northern China living in Beijing. Despite these limitations, our study provided some confirmation of the association of long-term TyG index patterns with incident CKD among non-diabetic population and further studies with different ages, regions and ethnic groups are still needed to validate our results.

## Conclusions

Overall, during a median of 3.82 years follow-up, we found that TyG index variability and cumulative TyG index can both increase the risk of incident CKD among non-diabetic population. Besides, extending baseline TyG index, TyG index variability and cumulative TyG index to the latest sex-specific risk predictive models for incident CKD showed the highest incremental effect. The current increasing ease and accuracy of collecting electronic health information by health systems provides a feasible and theoretical basis for assessing the long-term impact of TyG index, and we therefore believe that this study has substantial clinical implications and can provide basis for reducing the incidence and subsequent progression of CKD among non-diabetic population.

### Electronic supplementary material

Below is the link to the electronic supplementary material.

Supplementary Material 1

## Data Availability

The datasets used and/or analysed during the current study are available from the corresponding author on reasonable request.

## Abbreviations

CKD: chronic kidney disease
GBD: Global Burden of Disease
IR: insulin resistance
TyG: triglyceride glucose index
TG: triglyceride
FBG: fasting plasma glucose
T2D: type 2 diabetes
LDL-C: low-density lipoprotein cholesterol
HDL-C: high-density lipoprotein cholesterol
AST: aspartate aminotransferase
ALT: alanine aminotransferase
Cr: creatinine
UA: uric acid
HbA1c: Hemoglobin A1c
CV: coefficient of variation
SD: standard deviation
ARV: average real variability
VIM: variability independent of the mean
eGFR: estimate glomerular fltration rate
KDIGO: Kidney Disease:Improving Global Outcomes
CKD-EPI: Chronic Kidney Disease Epidemiology Collaboration
BMI: body mass index
BP: blood pressure
SBP: systolic blood pressure
DBP: diastolic blood pressure
ICC: intraclass correlation coefficient
HR: hazard ratio
CI: confidence intervals
RCS: restricted cubic spline
RERI: relative excess risk due to interaction
AP: attributable proportion due to interaction
NRI: net reclassification improvement
IDI: integrated discrimination improvement
IL: interleukin
TNF: tumor necrosis factor
ROS: reactive oxidative stress

## References

[1] Zhang LF, Wang L, Wang et al. Prevalence of chronic kidney disease in China: a cross-sectional survey. Lancet. 2012;379(9818):815 – 22.10.1016/s0140-6736(12)60033-6. 10.1016/S0140-6736(12)60033-6 22386035

[2] Xie YB, Bowe AH, Mokdad (2018). Analysis of the Global Burden of Disease study highlights the global, regional, and national trends of chronic Kidney Disease epidemiology from 1990 to 2016. Kidney Int.

[3] Gansevoort RTK, Matsushita M, van der Velde (2011). Lower estimated GFR and higher albuminuria are associated with adverse kidney outcomes. A collaborative meta-analysis of general and high-risk population cohorts. Kidney Int.

[4] Go ASGM, Chertow D, Fan (2004). Chronic Kidney Disease and the risks of death, cardiovascular events, and hospitalization. N Engl J Med.

[5] Caro JF (1991). Insulin resistance in obese and nonobese man. J Clin Endocrinol Metab.

[6] de Boer IH, Mehrotra R (2014). Insulin resistance in chronic Kidney Disease: a step closer to effective evaluation and treatment. Kidney Int.

[7] Ren XM, Jiang L, Han (2023). Association between triglyceride-glucose index and chronic Kidney Disease: a cohort study and meta-analysis. Nutr Metab Cardiovasc Dis.

[8] Cersosimo EC, Solis-Herrera ME, Trautmann (2014). Assessment of pancreatic β-cell function: review of methods and clinical applications. Curr Diabetes Rev.

[9] Sánchez-García AR, Rodríguez-Gutiérrez L, Mancillas-Adame et al. Diagnostic Accuracy of the Triglyceride and Glucose Index for Insulin Resistance: A Systematic Review. Int J Endocrinol. 2020;2020:4678526.10.1155/2020/4678526. 10.1155/2020/4678526 PMC7085845 32256572

[10] Simental-Mendía LE, Rodríguez-Morán M, Guerrero-Romero F (2008). The product of fasting glucose and triglycerides as surrogate for identifying insulin resistance in apparently healthy subjects. Metab Syndr Relat Disord.

[11] Shi YL, Hu M, Li (2022). Association between the Surrogate Markers of Insulin Resistance and chronic Kidney Disease in Chinese hypertensive patients. Front Med (Lausanne).

[12] Liu NC, Liu Z, Qu (2023). Association between the triglyceride-glucose index and chronic Kidney Disease in adults. Int Urol Nephrol.

[13] Pan XF, Hong S, Li (2023). Long-term exposure to ambient PM(2.5) constituents is associated with dyslipidemia in Chinese adults. Ecotoxicol Environ Saf.

[14] Mao T, Akshit FNU, Mohan MS (2023). Effects of anthocyanin supplementation in diet on glycemic and related cardiovascular biomarkers in patients with type 2 Diabetes: a systematic review and meta-analysis of randomized controlled trials. Front Nutr.

[15] Qi SY, Xu K, Zeng (2023). Incidence and Factors Associated with Hyperglycemia in patients with first hospitalization for Major Depression disorder: a large cross-sectional sample. Neuropsychiatr Dis Treat.

[16] Tai SL, Fu N, Zhang (2022). Association of the cumulative triglyceride-glucose index with major adverse cardiovascular events in patients with type 2 Diabetes. Cardiovasc Diabetol.

[17] Li HY, Zuo F, Qian (2022). Triglyceride-glucose index variability and incident Cardiovascular Disease: a prospective cohort study. Cardiovasc Diabetol.

[18] Yan YXJ, Dong YQ, Liu (2012). Association of suboptimal health status and cardiovascular risk factors in urban Chinese workers. J Urban Health.

[19] Kim MKK, Han YM, Park (2018). Associations of variability in blood pressure, glucose and cholesterol concentrations, and body Mass Index with Mortality and Cardiovascular outcomes in the General Population. Circulation.

[20] Wang XB, Feng Z, Huang (2022). Relationship of cumulative exposure to the triglyceride-glucose index with ischemic Stroke: a 9-year prospective study in the Kailuan cohort. Cardiovasc Diabetol.

[21] Li CY, Zhu Y, Ma (2022). Association of cumulative blood pressure with Cognitive decline, Dementia, and Mortality. J Am Coll Cardiol.

[22] Andrassy KM (2013). Comments on ‘KDIGO 2012 Clinical Practice Guideline for the evaluation and management of chronic kidney. Disease’ Kidney Int.

[23] Levey ASL, A. Stevens CH, Schmid et al. A new equation to estimate glomerular filtration rate. Ann Intern Med. 2009;150(9):604 – 12.10.7326/0003-4819-150-9-200905050-00006. 10.7326/0003-4819-150-9-200905050-00006 PMC2763564 19414839

[24] Lee SM, Kim SH, Yoon HJ (2023). Prediction of incident chronic Kidney Disease in a population with normal renal function and normo-proteinuria. PLoS ONE.

[25] Lei LH, Liang Y, Qu (2022). Association between triglyceride-glucose index and worsening renal function in the elderly. Front Nutr.

[26] Gao WJ, Wang Y, Chen (2021). Discordance between the triglyceride glucose index and HOMA-IR in incident albuminuria: a cohort study from China. Lipids Health Dis.

[27] Zhu QY, Chen X, Cai (2022). The non-linear relationship between triglyceride-glucose index and risk of chronic Kidney Disease in hypertensive patients with abnormal glucose metabolism: a cohort study. Front Med (Lausanne).

[28] Liu LZ, Wu Y, Zhuang (2022). Association of triglyceride-glucose index and traditional risk factors with Cardiovascular Disease among non-diabetic population: a 10-year prospective cohort study. Cardiovasc Diabetol.

[29] Wang DW, Li M, Zhou (2023). Association of the triglyceride-glucose index variability with blood pressure and Hypertension: a cohort study. QJM.

[30] Affret AS, Wagner D, El, Fatouhi (2017). Validity and reproducibility of a short food frequency questionnaire among patients with chronic Kidney Disease. BMC Nephrol.

[31] Zheng LD, Zhu Y, Xiao (2023). Microneedle coupled epidermal sensor for multiplexed electrochemical detection of Kidney Disease biomarkers. Biosens Bioelectron.

[32] Kress SP, Bramlage RW, Holl (2023). Validation of a risk prediction model for early chronic Kidney Disease in patients with type 2 Diabetes: data from the German/Austrian Diabetes prospective follow-up registry. Diabetes Obes Metab.

[33] Sim RCW, Chong NK, Loganadan (2023). Comparison of a chronic Kidney Disease predictive model for type 2 Diabetes Mellitus in Malaysia using Cox regression versus machine learning approach. Clin Kidney J.

[34] Schonmann Y (2022). Cardiovascular risk assessment: baseline snapshots or accumulated burden?. Eur J Prev Cardiol.

[35] Chen JRP, Wildman LL, Hamm (2004). Association between inflammation and insulin resistance in U.S. nondiabetic adults: results from the Third National Health and Nutrition Examination Survey. Diabetes Care.

[36] Guo WY, Song Y, Sun (2022). Systemic immune-inflammation index is associated with diabetic Kidney Disease in type 2 Diabetes Mellitus patients: evidence from NHANES 2011–2018. Front Endocrinol (Lausanne).

[37] Weisberg SPD, McCann M, Desai (2003). Obesity is associated with macrophage accumulation in adipose tissue. J Clin Invest.

[38] Bolton CHLG, Downs JG, Victory (2001). Endothelial dysfunction in chronic Renal Failure: roles of lipoprotein oxidation and pro-inflammatory cytokines. Nephrol Dial Transplant.

[39] Schrauben SJC, Jepson JY, Hsu (2019). Insulin resistance and chronic Kidney Disease progression, cardiovascular events, and death: findings from the chronic renal insufficiency cohort study. BMC Nephrol.

[40] Vázquez LAF, Pazos JR, Berrazueta (2005). Effects of changes in body weight and insulin resistance on inflammation and endothelial function in morbid obesity after bariatric Surgery. J Clin Endocrinol Metab.

[41] Cersosimo E, DeFronzo RA (2006). Insulin resistance and endothelial dysfunction: the road map to Cardiovascular Diseases. Diabetes Metab Res Rev.

