# Supplementary material for: Association of long-term triglyceride-glucose index patterns with the incidence of chronic kidney disease among non-diabetic population: evidence from a functional community cohort
Source: Cardiovasc Diabetol. 2024 Jan 3;23:7. doi: 10.1186/s12933-023-02098-7 (PMC10765660; doi:10.1186/s12933-023-02098-7)
Supplement: Supplementary file 1 — Supplementary Material 1 [file 12933_2023_2098_MOESM1_ESM.docx]

**Additional file 1**

1. Calculation of average real variability and variability independent of the mean

**Fig. S1** Flow chart of this study

**Table S1** Baseline characteristics of participants included and excluded from this study

**Table S2** Intraclass correlation coefficient of repeated TyG index measurements

**Table S3** Baseline characteristics of participants according to quartiles of TyG index variability

**Table S4** Baseline characteristics of participants according to quartiles of cumulative TyG index

**Fig. S2** Incidence of CKD among different groups

**Fig. S3** Dose-response relationship of incident CKD associated with continuous TyG index variability and cumulative TyG index after adjusting for potential confounders

**Table S5** Sensitivity analysis: Association of quartiles of TyG index variability or cumulative TyG index with the incidence of CKD

**Table S6** Sensitivity analysis: Association of quartiles of TyG index variability with the incidence of CKD by using standard deviation, average real variability and variability independent of the mean to determine the variability of TyG index

1. Calculation of average real variability and variability independent of the mean
2. average real variability (ARV)

ARV is the average of the absolute differences between consecutive values and was calculated using the following formula, where N denotes the number of measurements of the metabolic parameters. As such, $ARV=\frac{1}{N-1}\sum_{k=1}^{N-1} \left|Value_{k+1}-Value_{k}\right|$. In this study, N=3.

1. variability independent of the mean (VIM)

VIM is calculated first as the SD of TyG index divided by the mean TyG index raised to the power of x, where x is obtained from fitting a nonlinear regression model among the entire sample where $SD=a*mean^{x}$. This quantity is then multiplied by the sample mean TyG index raised to the power of x. As such, $VIM=\frac{k*SD(TyG)}{Mean(TyG)}$. Where, $k=Mean\left(Mean\left(TyG\right)\right)^{x}$.


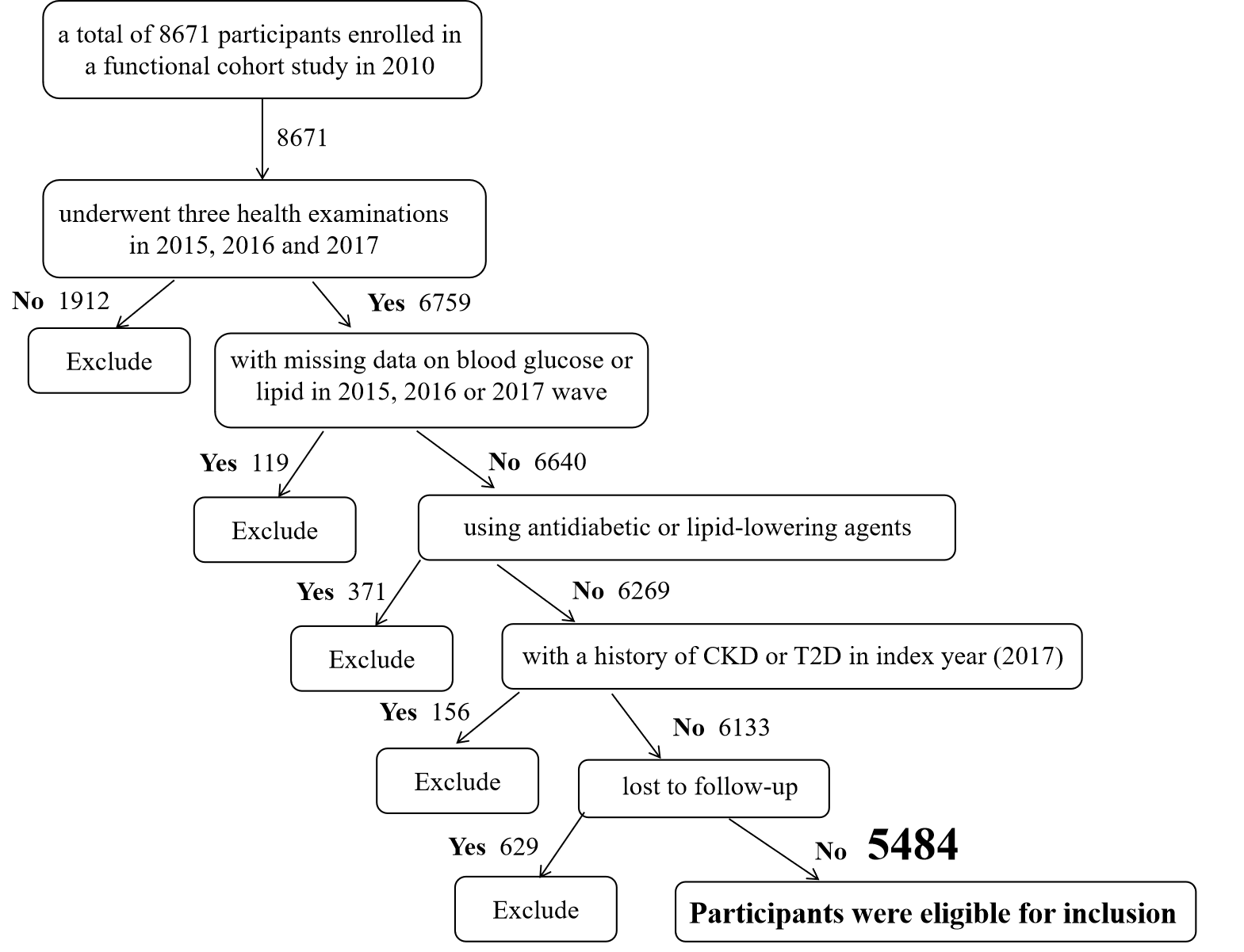


Fig. S1 Flow chart of this study

Table S1 Baseline characteristics of participants included and excluded from this study

|  | **Included**  N=5484 | **Excluded**  N=1275 | P |
| --- | --- | --- | --- |
| Age, y | 52.49±14.67 | 46.19±10.59 | < 0.001 |
| Men, n (%) | 2486 (45.33) | 555 (43.53) | 0.244 |
| Smoking, n (%) | 343 (6.25) | 100 (7.84) | 0.039 |
| Drinking, n (%) | 673 (12.27) | 183 (14.35) | 0.044 |
| Physical activity, n (%) | 3982 (72.61) | 877 (68.78) | 0.006 |
| Education, n (%) |  |  | 0.914 |
| Primary school  and below | 155 (2.83) | 36 (2.82) |  |
| Junior high school | 1442 (26.29) | 324 (25.41) |  |
| High school and above | 3887 (70.88) | 900 (70.59) |  |
| Hypertension, n (%) | 1306 (23.81) | 379 (29.73) | < 0.001 |
| SBP, mmHg | 123 [112, 136] | 127 [114, 140] | < 0.001 |
| DBP, mmHg | 75 [68, 82] | 77 [70, 84] | < 0.001 |
| BMI, kg/m2 | 24.22 [22.03, 26.56] | 25.22 [22.77, 27.73] | < 0.001 |
| AST, U/L | 19 [14, 26] | 20 [17, 24] | < 0.001 |
| ALT, U/L | 21 [18, 25] | 16 [12, 24] | < 0.001 |
| TG, mg/dL | 114.29 [80.63, 163.25] | 112.49 [75.29, 165.63] | 0.267 |
| LDL-C, mg/dL | 112.89 [93.17, 134.25] | 109.55 [88.55, 133.22] | 0.001 |
| HDL-C, mg/dL | 54.90 [46.01, 64.95] | 63.31 [53.86, 75.02] | < 0.001 |
| Glu, mg/dL | 93.78 [88.38, 100.08] | 101.88 [90.45, 113.04] | < 0.001 |
| HbA1c, % | 5.3 [5.0, 5.6] | 5.8 [5.5, 6.2] | < 0.001 |
| Urea, mmol/L | 4.91 [4.15, 5.85] | 4.93 [4.04, 5.84] | 0.088 |
| UA, μmol/L | 314 [263, 374] | 295 [234, 358] | < 0.001 |
| Cr, mg/dL | 0.72 [0.63, 0.85] | 0.66 [0.57, 0.79] | < 0.001 |
| eGFR, ml/min/1.73 m2 | 102.23 [93.16, 111.15] | 109.29 [100.57, 117.72] | < 0.001 |
| Baseline TyG | 8.60 [8.23, 8.97] | 8.67 [8.20, 9.08] | 0.006 |

SBP: systolic blood pressure, DBP: diastolic blood pressure, BMI: body mass index, TG: triglyceride; AST: aspartate aminotransferase; ALT: alanine aminotransferase; Cr: creatinine; UA: uric acid; Glu: plasma glucose, LDL-C: low-density lipoprotein cholesterol, HDL-C: high-density lipoprotein cholesterol, TyG index: triglyceride glucose index

Table S2 Intraclass correlation coefficient of repeated TyG index measurements

| TyG index | Median [quartiles] | ICC (95% CI) | *F* | *P* |
| --- | --- | --- | --- | --- |
| 2015 | 8.46 [8.09, 8.85] | 0.768 (0.759, 0.777) | 10.933 | < 0.001 |
| 2016 | 8.55 [8.17, 8.93] |  |  |  |
| 2017 | 8.60 [8.23, 8.97] |  |  |  |

TyG index: triglyceride-glucose index, ICC: intraclass correlation coefficient, CI: confidence interval

Table S3 Baseline characteristics of participants according to quartiles of TyG index variability

|  | Q1  N=1372 | Q2  N=1370 | Q3  N=1372 | Q4  N=1370 | *P* |
| --- | --- | --- | --- | --- | --- |
| Age, year | 53.80±15.35 | 53.87±14.93 | 52.58±14.26 | 49.69±13.71 | <0.001 |
| Men, n% | 626 (45.63) | 634 (46.28) | 642 (46.79) | 584 (42.63) | 0.124 |
| Smoking, n% | 88 (6.41) | 73 (5.33) | 93 (6.78) | 89 (6.50) | 0.417 |
| Drinking, n% | 161 (11.73) | 164 (11.97) | 178 (12.97) | 170 (12.41) | 0.769 |
| Physical activity, n% | 998 (72.74) | 1024 (74.74) | 966 (70.41) | 994 (72.55) | 0.090 |
| Education, n% |  |  |  |  | 0.810 |
| Primary school  and below | 34 (2.48) | 40 (2.92) | 38 (2.77) | 43 (3.14) |  |
| Junior high school | 357 (26.02) | 363 (26.50) | 347 (25.29) | 375 (27.37) |  |
| High school and above | 981 (71.50) | 967 (70.58) | 987 (71.94) | 952 (69.49) |  |
| Hypertension, n% | 340 (24.78) | 342 (24.96) | 351 (25.58) | 273 (19.93) | 0.001 |
| SBP, mmHg | 123 [112, 135] | 124 [113, 137] | 124 [113, 137] | 121 [111, 133] | <0.001 |
| DBP, mmHg | 75 [68, 81] | 76 [69, 83] | 76 [68, 83] | 75 [68, 82] | 0.045 |
| BMI, kg/m^2^ | 21.19 [22.05, 26.70] | 24.24 [22.13, 26.48] | 24.45 [22.15, 26.56] | 24.04 [21.79, 26.50] | 0.218 |
| AST, U/L | 18 [14, 26] | 19 [14, 25] | 19 [14, 26] | 19 [14, 26] | 0.227 |
| ALT, U/L | 21 [18, 25] | 21 [18, 25] | 21 [18, 24] | 21 [18, 25] | 0.568 |
| TG, mg/dL | 111.19 [79.74, 158.59] | 113.41 [80.63, 159.48] | 114.29 [81.51, 164.80] | 119.61 [81.51, 174.32] | 0.002 |
| TC, mg/dL | 181.70 [162.37, 206.06] | 184.79 [162.76, 207.22] | 184.41 [164.69, 208.76] | 184.02 [161.60, 205.19] | 0.240 |
| LDL, mg/dL | 111.73 [92.78, 134.54] | 114.05 [94.81, 135.31] | 113.27 [95.39z, 135.31] | 111.15 [90.08, 131.06] | 0.015 |
| HDL, mg/dL | 54.12 [45.62, 66.11] | 55.09 [46.39, 54.95] | 55.28 [46.78, 64.95] | 54.12 [44.85, 63.40] | 0.090 |
| Glu, mg/dL | 93.51 [88.02, 100.08] | 94.14 [88.38, 99.72] | 93.96 [88.74, 100.26] | 93.78 [88.20, 100.44] | 0.426 |
| HbA1c, % | 5.3 [5.0, 5,6] | 5.3 [5.0, 5.6] | 5.3 [5.0, 5.6] | 5.3 [5.0, 5.5] | 0.115 |
| Urea, mmol/L | 4.97 [4.21, 5.88] | 4.91 [4.16, 5.80] | 4.93 [4.17, 5.97] | 4.84 [4.04, 5.72] | 0.006 |
| UA, μmol/L | 311 [261, 373] | 315 [264, 373.75] | 315 [268, 375] | 313.5 [261.75, 373.75] | 0.657 |
| Cr, μmol/L | 64 [56, 75] | 65 [56, 75] | 64 [55, 75] | 63 [56, 75] | 0.323 |
| eGFR, ml/min/1.73 m^2^ | 101.15 [91.80, 110.39] | 101.23 [91.85, 109.83] | 102.14 [92.86, 111.10] | 104.35 [96.02, 112.80] | <0.001 |
| Baseline TyG index | 8.57 [8.21, 8.92] | 8.59 [8.23, 8.94] | 8.59 [8.23, 8.98] | 8.64 [8.24, 9.03] | 0.003 |
| Cumulative TyG index | 17.04 [16.24, 17.90] | 17.05 [16.16, 17.82] | 17.05 [16.23, 17.86] | 17.08 [16.19, 17.97] | 0.634 |
| TyG index variability | 0.011 [0.007, 0.014] | 0.021 [0.019, 0.023] | 0.031 [0.028, 0.034] | 0.047 [0.041, 0.055] | <0.001 |

CKD: chronic kidney disease, SBP: systolic blood pressure, DBP: diastolic blood pressure, BMI: body mass index, TG: triglyceride; TC: total cholesterol; AST: aspartate aminotransferase; ALT: alanine aminotransferase; Cr: creatinine; UA: uric acid; Glu: plasma glucose, LDL-C: low-density lipoprotein cholesterol, HDL-C: high-density lipoprotein cholesterol, TyG index: triglyceride glucose index

Table S4 Baseline characteristics of participants according to quartiles of cumulative TyG index

|  | Q1  N=1371 | Q2  N=1371 | Q3  N=1371 | Q4  N=1371 | *P* |
| --- | --- | --- | --- | --- | --- |
| Age, year | 46.47±14.62 | 53.00±14.84 | 55.91±14.10 | 54.47±13.28 | <0.001 |
| Men, n% | 495 (36.11) | 585 (42.67) | 687 (50.11) | 719 (52.44) | <0.001 |
| Smoking, n% | 79 (5.76) | 88 (6.42) | 89 (6.49) | 87 (6.35) | 0.854 |
| Drinking, n% | 158 (11.52) | 159 (11.60) | 187 (13.64) | 169 (12.33) | 0.299 |
| Physical activity, n% | 981 (71.55) | 986 (71.92) | 1000 (72.94) | 1015 (74.03) | 0.463 |
| Education, n% |  |  |  |  | 0.711 |
| Primary school  and below | 40 (2.92) | 34 (2.48) | 41 (2.99) | 40 (2.92) |  |
| Junior high school | 356 (25.97) | 342 (24.95) | 378 (27.57) | 366 (26.70) |  |
| High school and above | 975 (71.12) | 995 (72.57) | 952 (69.44) | 965 (70.39) |  |
| Hypertension, n% | 170 (12.40) | 297 (21.66) | 413 (30.12) | 426 (31.07) | <0.001 |
| SBP, mmHg | 115 [106, 127] | 122[112,134.5] | 128 [116, 139] | 128 [117, 139] | <0.001 |
| DBP, mmHg | 71 [65, 78.75] | 75 [68, 81] | 77 [70, 84] | 78 [72, 85.25] | <0.001 |
| BMI, kg/m^2^ | 22.49 [20.70, 24.72] | 23.62 [21.75, 25.80] | 25.09 [22.94, 27.16] | 25.81 [23.56, 27.76] | <0.001 |
| AST, U/L | 16 [12, 21] | 17 [14, 23] | 20 [15, 27] | 22 [17, 32] | <0.001 |
| ALT, U/L | 20 [17, 23] | 21 [18, 24] | 22 [19, 25] | 22 [19, 27] | <0.001 |
| TG, mg/dL | 71.77 [56.70, 93.92] | 95.69 [78.85, 117.84] | 131.13 [106.32, 161.25] | 193.15 [147.08, 257.38] | <0.001 |
| TC, mg/dL | 175.52 [156.19, 194.85] | 179.77 [161.60, 203.74] | 186.73 [165.46, 208.96] | 194.07 [171.26, 217.66] | <0.001 |
| LDL, mg/dL | 102.45 [84.86, 120.23] | 112.11 [93.56, 130.48] | 118.69 [98.20, 140.34] | 120.62 [99.16, 143.43] | <0.001 |
| HDL, mg/dL | 62.63 [53.35, 73.07] | 57.60 [49.87, 67.66] | 52.96 [45.23, 61.08] | 4.78 [40.21, 54.90] | <0.001 |
| Glu, mg/dL | 90.54 [85.50, 95.76] | 92.88 [87.84, 98.46] | 95.22 [89.73, 101.52] | 97.02 [91.62, 104.22] | <0.001 |
| HbA1c, % | 5.1 [4.9, 5.4] | 5.3 [5.0, 5.6] | 5.4 [5.1, 5.6] | 5.4 [5.1, 5.7] | <0.001 |
| Urea, mmol/L | 4.68 [3.86, 5.65] | 4.87 [4.12, 5.79] | 5.05 [4.34, 5.96] | 5.05 [4.29, 5.90] | <0.001 |
| UA, μmol/L | 284 [239, 339] | 301 [256, 350] | 330 [280, 386] | 351 [294, 413] | <0.001 |
| Cr, μmol/L | 61 [54, 72] | 64 [55, 74] | 66 [57, 77] | 67 [57, 77] | <0.001 |
| eGFR, ml/min/1.73 m^2^ | 108.23 [99.33, 115.81] | 102.31 [93.35, 110.70] | 98.93 [90.59, 107.43] | 100.05 [92.06, 108.10] | <0.001 |
| Baseline TyG index | 8.10 [7.84, 8.37] | 8.40 [8.21, 8.62] | 8.74 [8.54, 8.95] | 9.17 [8.88, 9.44] | <0.001 |
| Cumulative TyG index | 15.59 [15.07, 15.92] | 16.65 [16.44, 16.86] | 17.45 [17.26, 17.67] | 18.52 [18.17, 19.20] | <0.001 |
| TyG index variability | 0.025 [0.016, 0.037] | 0.025 [0.016, 0.037] | 0.025 [0.016, 0.037] | 0.026 [0.016, 0.038] | 0.439 |

CKD: chronic kidney disease, SBP: systolic blood pressure, DBP: diastolic blood pressure, BMI: body mass index, TG: triglyceride; TC: total cholesterol; AST: aspartate aminotransferase; ALT: alanine aminotransferase; Cr: creatinine; UA: uric acid; Glu: plasma glucose, LDL-C: low-density lipoprotein cholesterol, HDL-C: high-density lipoprotein cholesterol, TyG index: triglyceride glucose index


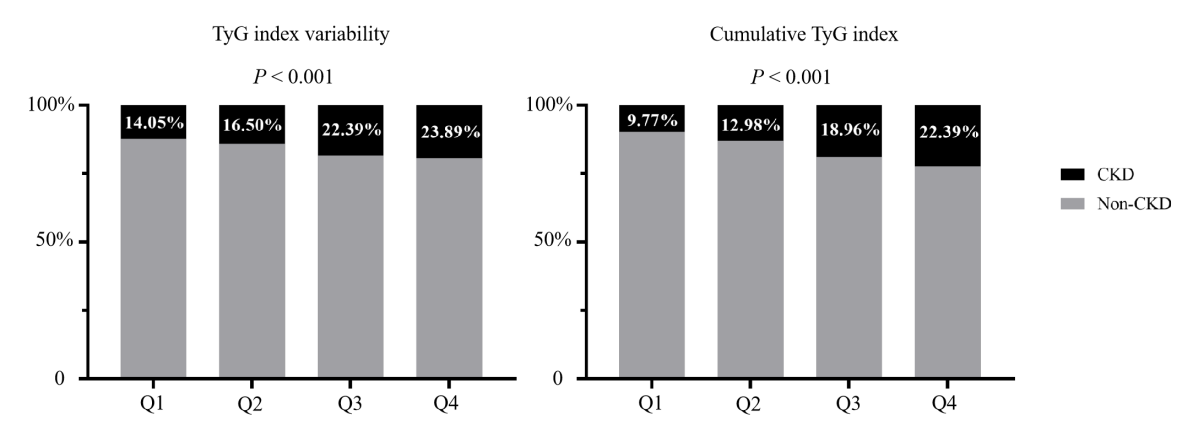


Fig. S2 Incidence of CKD among different groups


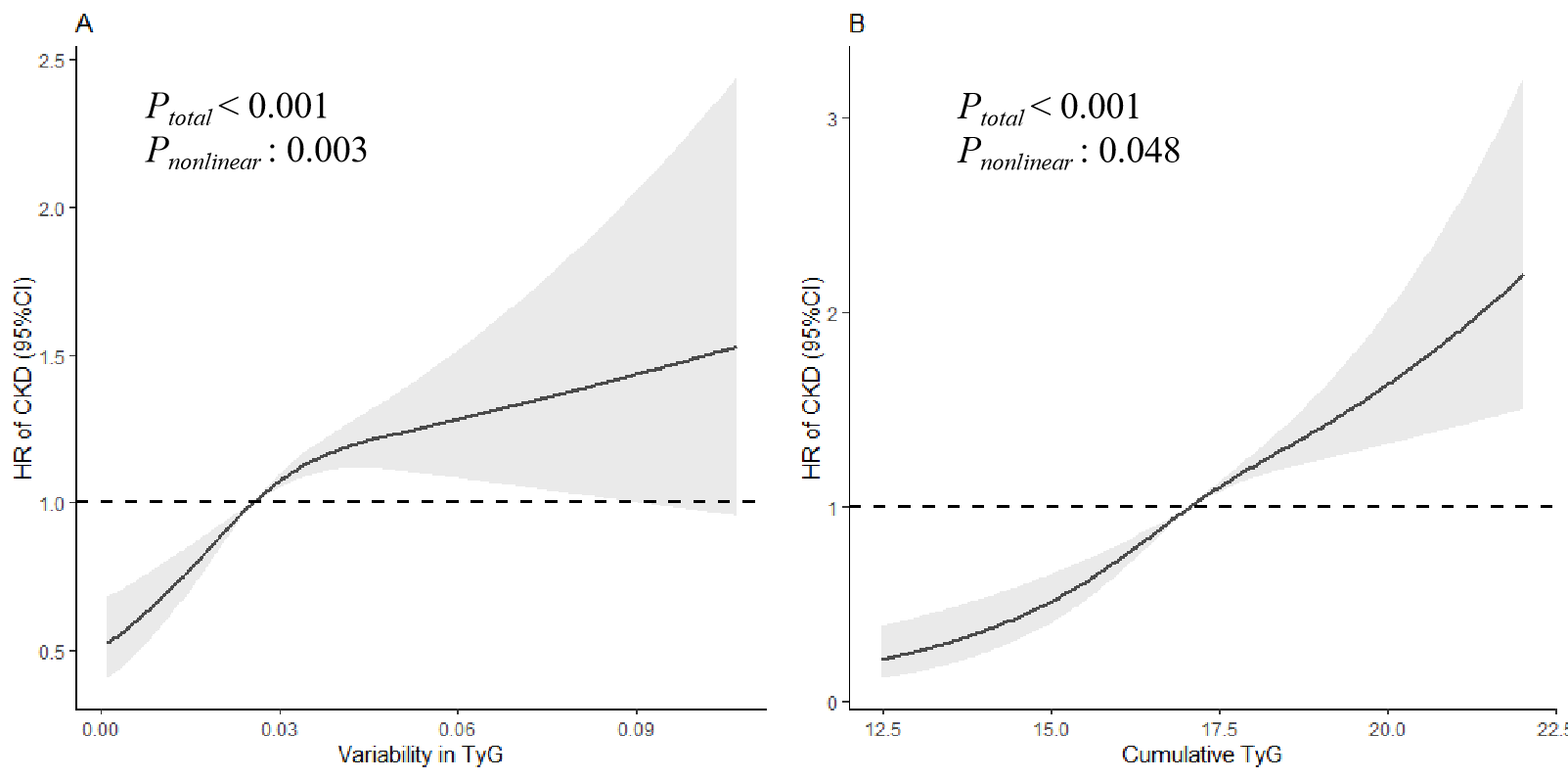


Fig. S3 Dose-response relationship of incident CKD associated with continuous TyG index variability and cumulative TyG index after adjusting for potential confounders

Table S5 Sensitivity analysis: Association of quartiles of TyG index variability or cumulative TyG index with the incidence of CKD

|  | **Sensitivity 1** |  | **Sensitivity 2** |  | **Sensitivity 3** |  |
| --- | --- | --- | --- | --- | --- | --- |
|  | HR (95% CI) | *P* | HR (95% CI) | *P* | HR (95% CI) | *P* |
| **TyG index variability** | |  |  |  |  |  |
| Q1 (Ref.) | - |  | - |  | - |  |
| Q2 | 1.239 (0.995, 1.542) | 0.055 | 1.192 (0.964, 1.475) | 0.106 | 1.081 (0.827, 1.413) | 0.570 |
| Q3 | 1.557 (1.266, 1.916) | < 0.001 | 1.515 (1.239, 1.853) | < 0.001 | 1.347 (1.049, 1.730) | 0.020 |
| Q4 | 1.817 (1.478, 2.232) | < 0.001 | 1.764 (1.446, 2.152) | < 0.001 | 1.722 (1.347, 2.200) | < 0.001 |
| *P* for trend |  | < 0.001 |  | < 0.001 |  | < 0.001 |
| **Cumulative TyG index** | |  |  |  |  |  |
| Q1(Ref.) | - |  |  |  |  |  |
| Q2 | 1.135 (0.890, 1.447) | 0.307 | 1.162 (0.915, 1.475) | 0.219 | 1.192 (0.890, 1.596) | 0.238 |
| Q3 | 1.663 (1.289, 2.145) | < 0.001 | 1.691 (1.310, 2.182) | < 0.001 | 1.464 (1.083, 1.979) | 0.013 |
| Q4 | 2.124 (1.589, 2.838) | < 0.001 | 2.223 (1.627, 3.307) | < 0.001 | 1.963 (1.427, 2.698) | < 0.001 |
| *P* for trend |  | < 0.001 |  | < 0.001 |  | < 0.001 |

CKD: chronic kidney disease, TyG index: triglyceride glucose index

Sensitivity 1: participants experiencing CKD within one year were excluded.

Sensitivity 2: the mean values of TyG from 2015 to 2017 instead of baseline values was adjusted in Model 3 of the Cox proportional-hazards model.

Sensitivity 3: participants with abnormal TG concentrations at baseline (≥1.7 mmol/L) were excluded

Table S6 Sensitivity analysis: Association of quartiles of TyG index variability with the incidence of CKD by using standard deviation, average real variability and variability independent of the mean to determine the variability of TyG index

|  | **SD** |  | **ARV** |  | **VIM** |  |
| --- | --- | --- | --- | --- | --- | --- |
|  | HR (95% CI) | *P* | HR (95% CI) | *P* | HR (95% CI) | *P* |
| **TyG index variability** | |  |  |  |  |  |
| Q1 (Ref.) | - |  | - |  | - |  |
| Q2 | 1.129 (0.911, 1.399) | 0.267 | 1.062 (0.856, 1.318) | 0.583 | 1.182 (0.955, 1.463) | 0.125 |
| Q3 | 1.420 (1.157, 1.743) | 0.001 | 1.475 (1.206, 1.805) | < 0.001 | 1.524 (1.247, 1.862) | < 0.001 |
| Q4 | 1.776 (1.455, 2.167) | < 0.001 | 1.643 (1.346, 2.005) | < 0.001 | 1.711 (1.400, 2.092) | < 0.001 |
| *P* for trend |  | < 0.001 |  | < 0.001 |  | < 0.001 |

CKD: chronic kidney disease, TyG index: triglyceride glucose index, SD: standard deviation, ARV: average real variability, VIM: variability independent of the mean
